# A Case of Mature Cystic Teratoma Arising from the Fourth Ventricle

**DOI:** 10.1155/2013/702424

**Published:** 2013-05-26

**Authors:** Parikshit Sanyal, Sanghita Barui, Smriti Mathur, Utpal Basak

**Affiliations:** Department of Pathology, Command Hospital (EC), Alipore, Kolkata 27, India

## Abstract

Intracranial teratomas represent a rare lesion accounting for 0.1%–0.7% of all intracranial tumors. Those in the fourth ventricle have rarely been reported. The present case is that of a 28-year-old man with occipital headache for two months. MRI examination revealed a well-defined extra-axial cystic lesion in posterior fossa in the midline herniating through the foramen magnum. Pre operatively, the mass was seen to be occupying the whole of the posterior fossa and arising from the roof of the fourth ventricle. On gross examination, the lesion had both solid and cystic components. Histopathological examination showed multiple cystic areas lined by brain tissue admixed with islands of cartilage and salivary gland elements and intestinal type glands. A diagnosis of mature cystic teratoma was made.

## 1. Introduction

Intracranial teratomas represent a rare lesion accounting for 0.1%–0.7% of all intracranial tumors. However, their location in the posterior fossa is uncommon. Most of intracranial teratomas have been found to occur in the midline structures, particularly the pineal and suprasellar regions; those in the fourth ventricle have rarely been reported [1]. 

## 2. Case History

**Figure 1:**
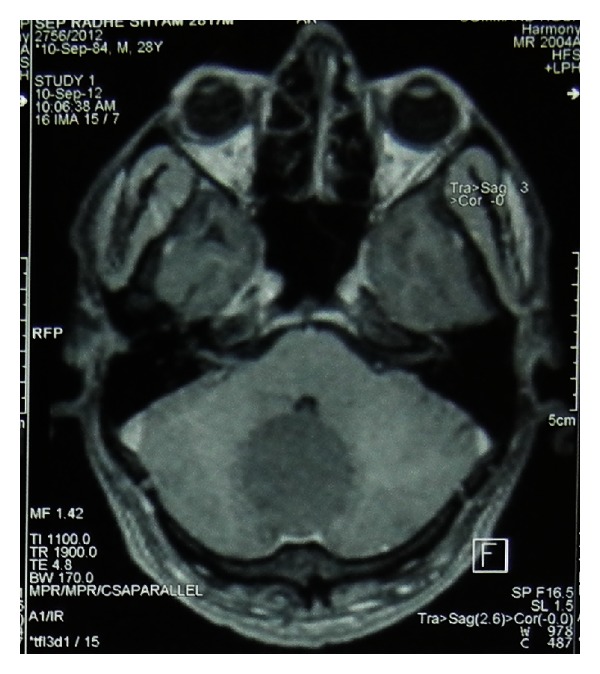
MRI (axial view) Showing mass in posterior fossa.

**Figure 2:**
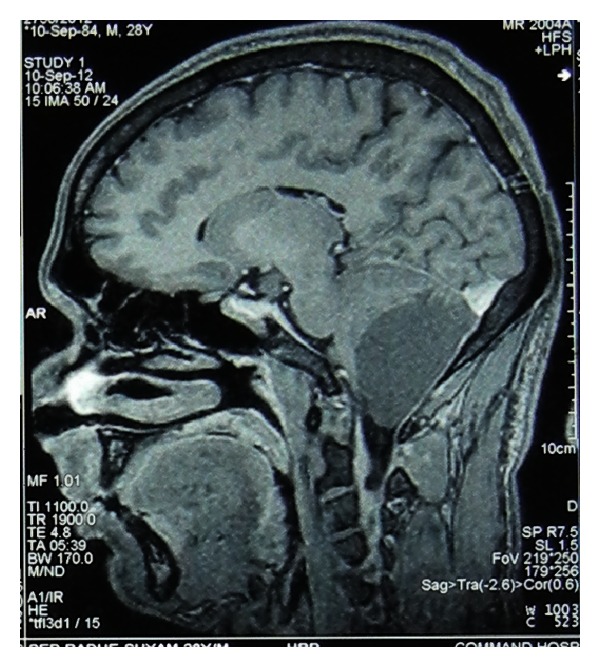
MRI (sagittal view) Showing mass in posterior fossa.

Our case, a 28-year-old man, presented with severe headache in occipital region for 2 months. On MRI examination, the mass was seen to be measuring 5.8 × 3.9 × 4.4 cm in size. It was a well-defined extra-axial cystic lesion located in the posterior fossa, herniating through the foramen magnum into the upper cervical canal and causing compression over the cervicomedullary junction (Figures 1 and 2).

The case was taken up for suboccipital craniotomy. Pre operatively, the mass was seen to be occupying the whole of the posterior fossa and arising from the roof of the fourth ventricle. The mass was completely excised and sent in pieces for histopathological examination.

On gross examination, it was noted to be in several bits, the largest measuring 2.8 × 2 cm in diameter. Few of the bits showed a cystic component. A few nodular, hard areas were also noted in some of the bits.

**Figure 3:**
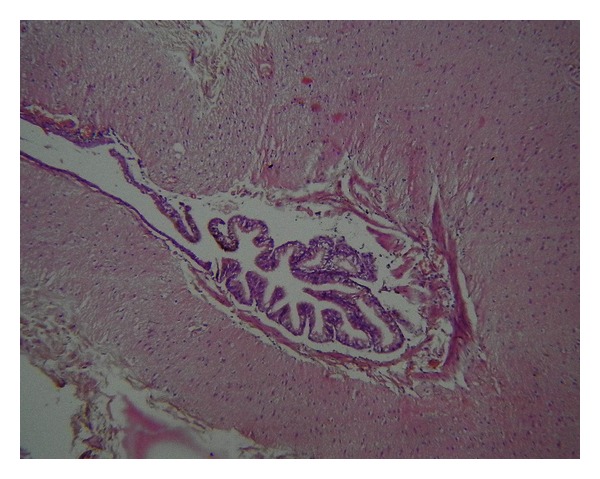
Section from the tumor showing intestinal type glands surrounded by cerebral parenchyma, H & E 10X objective.

**Figure 4:**
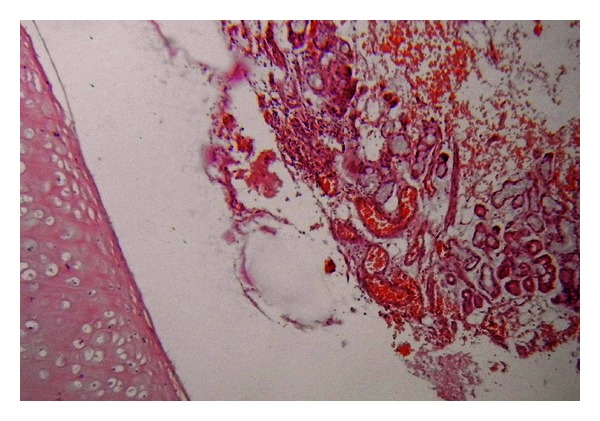
Section from the tumor showing cartilage tissue with salivary glands, H & E 10x objective.

**Figure 5:**
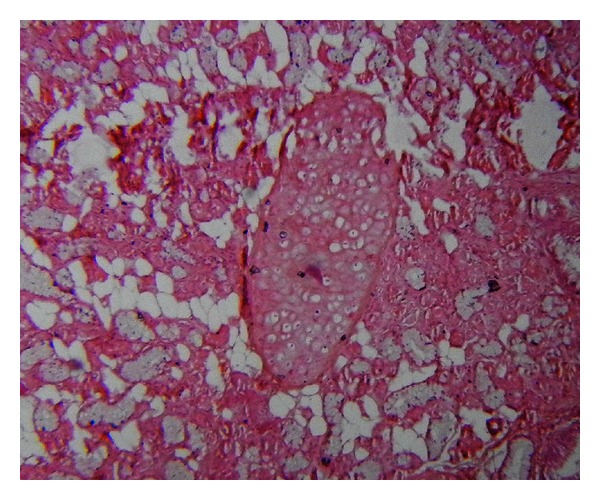
Section from tumor showing a cartilage island within salivary tissue, H & E 10x objective.

**Figure 6:**
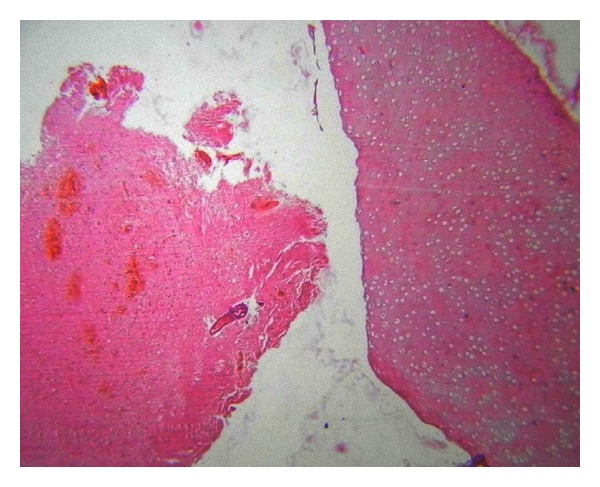
Section showing cartilage with brain tissue (H & E 4x objective).

Histopathological examination showed multiple cystic areas lined by brain tissue admixed with islands of cartilage (Figures 4, 5, and 6) and salivary gland (Figure 5) elements and occasional intestinal type glands (Figure 3). 

## 3. Discussion

Teratomas are nonseminomatous germ cell tumors differentiating into all three germ layers. However, all the layers may *not* be seen in every case of teratoma. In the classic study on ovarian teratomas, Blackwell et al. [2] found ectodermal derivatives in 100% of the tumors, mesodermal structures in 93%, and endodermal derivatives in 71%. 

Intracranial mature teratomas are tumors with a very low incidence (0.2%) and a clear male predominance (5 : 1). This incidence is higher in children. Most of the reported intracranial teratomas were located in the supratentorial midline and the pineal region. We report the case of a 26-year-old man with a mature teratoma in the midline posterior fossa arising from the fourth ventricle.

Definitive diagnosis was achieved by means of histological study, when cartilage, salivary gland tissue, and intestinal type glands were identified adjacent to brain tissue in the tumor. However, no ectodermal derivatives like skin, teeth, hair, and so forth, were found. No element of immature neural tissue was found as well.

To our knowledge, only six cases of mature teratomas in the fourth ventricle have been reported in detail in the literature to date [3–8]. One of them was a “double teratoma” of the pineal region and the fourth ventricle [8]. The largest series of intracranial pediatric teratomas reported by Noudel et al. revealed pure mature teratomas in 5 out of 14 patients. However, all of them were located in the sellar or the pineal regions and none of them in the posterior fossa [9]. 

From India, a radiographic report was published by Morelli [4], and the first histologically confirmed case was reported by Blackwell et al. [2]. We found that the present case was only the second mature teratoma of the fourth ventricle reported from India which has been histologically confirmed.
